# Recurrence monitoring using ctDNA in patients with metastatic colorectal cancer: COSMOS-CRC-03 and AURORA studies

**DOI:** 10.1016/j.esmogo.2023.100034

**Published:** 2024-02-03

**Authors:** E. Oki, R. Nakanishi, K. Ando, I. Takemasa, J. Watanabe, N. Matsuhashi, T. Kato, Y. Kagawa, M. Kotaka, K. Hirata, M. Sugiyama, T. Kusumoto, Y. Miyamoto, K. Toyosaki, J. Kishimoto, Y. Kimura, T. Yoshizumi, Y. Nakamura

**Affiliations:** 1 Department of Surgery and Science, Graduate School of Medical Sciences, Kyushu University, Fukuoka; 2 Department of Surgery, Surgical Oncology and Science, Sapporo Medical University, Sapporo; 3 Department of Surgery, Gastroenterological Center, Yokohama City University Medical Center, Yokohama; 4 Department of Gastrointestinal Surgery, Gifu University Hospital, Gifu; 5 Department of Surgery, National Hospital Organization Osaka National Hospital, Osaka; 6 Department of Gastrointestinal Surgery, Osaka General Medical Center, Osaka; 7 Gastrointestinal Cancer Center, Sano Hospital, Kobe; 8 Department of Surgery 1, School of Medicine, University of Occupational and Environmental Health, Kitakyushu; 9 Gastroenterological Surgery, National Hospital Organization Kyushu Cancer Center, Fukuoka; 10 Department of Gastroenterological Surgery, Clinical Research Center, Cancer Research Division, National Hospital Organization Kyushu Medical Center, Fukuoka; 11 Department of Gastroenterological Surgery, Graduate School of Medical Sciences, Kumamoto University, Kumamoto; 12 Center for Clinical and Translational Research, Kyushu University Hospital, Fukuoka; 13 Translational Research Support Section, National Cancer Center Hospital East, Kashiwa, Japan

**Keywords:** adjuvant chemotherapy, colorectal cancer, ctDNA, liver metastasis, oligometastasis, mFOLFOXIRI plus bevacizumab

## Abstract

International treatment guidelines recommend tumor resection for patients with oligometastatic colorectal cancer (CRC). Despite this, recurrence occurs in ∼60% of patients post-surgery, indicating that the role and optimal type of perioperative systemic therapy has not been fully defined. In the COSMOS-oligo trials, comprising two studies, we are evaluating the potential role of circulating tumor DNA (ctDNA) analysis in clinical decision making and exploring adjuvant therapy strategies for patients with resectable metastatic CRC. The COSMOS-CRC-03 study aims to evaluate the prognostic value of post-operative minimal residual disease as detected by ctDNA and to explore the role of ctDNA in detecting disease recurrence. We plan to assess the predictive accuracy of ctDNA results for recurrence using blood collected 28 days post-surgery. We will additionally explore whether regular post-operative ctDNA test can detect recurrence earlier than standard imaging. Post-operative adjuvant therapy will not be administered to ctDNA-negative patients. The complementary AURORA trial is a randomized phase II study designed to test whether post-operative mFOLFOXIRI plus bevacizumab is superior to standard mFOLFOX6 for patients with metastatic CRC when the ctDNA status is positive after curative-intent surgery for patients enrolled in the COSMOS-CRC-03 study. Both studies will only include patients with resectable distant metastases of CRC. We designed these studies to stratify patients based on the results of a ctDNA assay and to determine the optimal treatment for patients at the highest risk for recurrence.

## Highlights

• Treatment guidelines recommend resection of oligometastatic CRC.
• Optimal perioperative systemic therapy has not been fully defined.
• The COSMOS-CRC-03 study evaluates the ctDNA’s prognostic value for oligometastatic cancer.
• The AURORA trial tests mFOLFOXIRI plus bevacizumab post-surgery.
• We designed these studies to stratify patients based on a ctDNA assay.

## Introduction

Numerous clinical trials of adjuvant chemotherapy for colorectal cancer (CRC) have been conducted worldwide, and a combination of surgery and appropriate adjuvant chemotherapy has been proven effective in managing stage I-III CRC.^1^^,^^2^ Resection is also considered for stage IV disease if distant metastases are resectable. However, even with complete resection of distant metastases, recurrence is observed in >60% of patients.^3^^,^^4^ Furthermore, treatment strategies for perioperative chemotherapy for resection of metastases are not as well established as those for stage II-III cases. Even after resection of liver metastases, which is the most frequent site for distant disease, the necessity for perioperative adjuvant chemotherapy is controversial.^4^^,^^5^

Recently, the use of circulating tumor DNA (ctDNA), which is released from cancer cells into the bloodstream, has been reported to be particularly useful as a prognostic tool for CRC.^6^, ^7^, ^8^, ^9^ Several large-scale prospective clinical randomized trials are currently being conducted to evaluate adjuvant chemotherapy and treatment regimens using ctDNA as a biomarker.^10^, ^11^, ^12^ However, few has focused exclusively on stage IV cases in which distant metastases are resected.^13^

This study program, focusing on patients with oligometastatic CRC, aims to determine the utility of ctDNA as a potential tool for risk stratification after tumor resection and for early detection of recurrence. A randomized phase II trial will be conducted simultaneously to explore the clinical value of two adjuvant chemotherapy regimens for patients with minimal residual disease (MRD) after surgery.

## Protocol design

**Figure 1:**
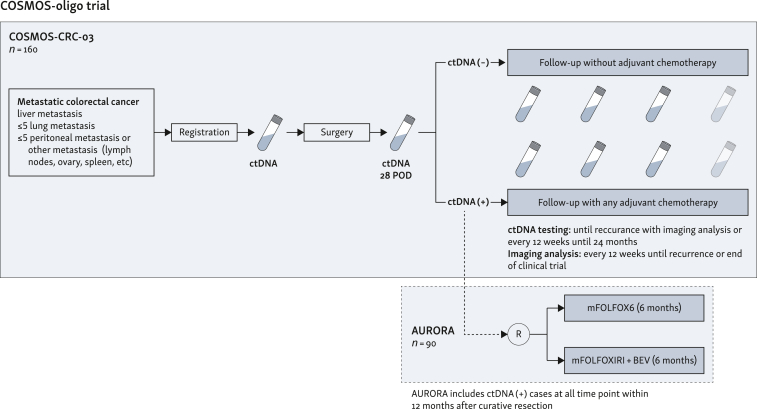
**COSMOS-oligometastasis overview.** BEV, bevacizumab; ctDNA, circulating tumor DNA; POD, post-operative days.

The COSMOS-oligo program consists of two clinical studies: COSMOS-CRC-03 and AURORA. The AURORA trial is designed for ctDNA-positive cases identified in the COSMOS-CRC-03 study (Figure 1).

These two trials will utilize a tumor-agnostic, blood-only ctDNA assay (Guardant Reveal, Guardant Health, Redwood City, CA). This assay is a next-generation sequencing test validated in stage I-III CRC that detects the presence of DNA methylation signatures associated with CRC. COSMOS-CRC-03 is designed to establish the clinical significance of stratifying a patient population into high and low risks of recurrence using ctDNA. Patients with detectable ctDNA in blood collected 28 days post-surgery will receive adjuvant chemotherapy and be encouraged to do so by participating in the complementary AURORA trial. Patients whose post-operative blood samples are negative for ctDNA will not receive immediate chemotherapy, but will be monitored by ctDNA testing and imaging every 12-24 weeks for up to 2 years. When ctDNA results become positive or imaging suggests disease recurrence, patients will be offered chemotherapy outside of the AURORA study.

## COSMOS-CRC-03

### Intervention

COSMOS-CRC-03 is a single-arm, open-label, interventional trial where the ctDNA status post-surgical resection of oligometastatic CRC determines the use of adjuvant chemotherapy. If ctDNA is detected after resection, patients will receive chemotherapy; if ctDNA is not detected, immediate chemotherapy will not be administered. In this trial, we will investigate the clinical significance of ctDNA under the following hypotheses: (i) the absence of ctDNA at post-operative day 28 can be used to select a population with a good prognosis after resection of distant metastases of CRC, and (ii) ctDNA testing every 12-24 weeks (up to 12 months) can detect recurrence earlier than standard imaging tests. Detailed inclusion and exclusion criteria are listed in Table 1.

**Table 1:** COSMOS-CRC-03 criteria

Inclusion criteria
1. The patient was diagnosed with colorectal cancer based on histological diagnosis of the primary lesion.
2. The following metastatic lesions have been clinically diagnosed as colorectal cancer (regardless of whether they are synchronous or metachronous): (i) lung metastasis (<5 in preoperative diagnosis) (ii) liver metastasis (any number) (iii) peritoneal metastasis (<5 in preoperative diagnosis) (iv) extra-regional lymph node metastasis (<3 in preoperative diagnosis) (v) ovarian metastasis (vi) other metastases such as adrenal or splenic metastases (confirm with coordinating physician before enrollment) Metastases involving multiple organs are also eligible.
3. The distant metastases are diagnosed by one of the following tests: (i) Chest computed tomography (CT) (5 mm slices) (both plain and contrast-enhanced CT are acceptable) (ii) Upper abdominal contrast-enhanced CT (5 mm slices)^a^ or upper abdominal magnetic resonance imaging (MRI) (5 mm slices) (iii) Pelvic contrast-enhanced CT (5 mm slices)^a^ or pelvic MRI (5 mm slices) ^a^If the patient is allergic to contrast agent, plain CT is acceptable.
4. Surgical resection is planned for the primary and metastatic lesions. One-stage surgery should be carried out for distant metastases. However, two-stage surgery within 28 days is acceptable in cases of metastasis in the right or left lung. Either of the following cases are eligible: (i) R0 resection is planned for the primary lesion and distant metastases. (Resection of the primary and metastatic lesions as a two-step surgery within 28 days is permitted.) (ii) R0 resection has been carried out for the primary lesion, and the first R0 resection for a distant lesion is scheduled. (iii) R0 resection has been carried out for the primary and metastatic lesion, and the R0 resection for a distant lesion is scheduled.
5. Patients will be eligible only if the history of chemotherapy or radiotherapy meets either of the conditions (i) or (ii) below. (i) At the time of enrollment, 28 days have passed since the final infusion of chemotherapy. (ii) Preoperative radiotherapy was administered for the primary or previous metastatic lesion.
6. If metastases to the liver or lungs are present, the patient has no history of cryotherapy or thermocoagulation therapy such as radiofrequency cauterization (including concomitant administration at the time of resection of metastases to the liver).
7. Being aged ≥18 years at the time of obtaining written consent.
8. Eastern Cooperative Oncology Group performance status (PS) of 0 or 1. For patients aged ≥75 years, only a PS 0 is acceptable.
9. The patient is able to have blood collected according to the protocol for this study after enrollment.
10. The patient has fulfilled all of the following test findings: (The latest test results within the period of 14 days before enrollment should be used. The day 1 week before the enrollment is acceptable.) (i) Neutrophil count ≥1500/mm^3^ (ii) Platelet count ≥10 × 10^4^/mm^3^ (iii) AST ≤100 IU/l (for liver metastases, ≤150 IU/l allowed) (iv) ALT ≤100 IU/l (for liver metastases, ≤150 IU/l allowed) (v) Serum total bilirubin ≤1.5 mg/dl (vi) Serum creatinine ≤1.4 mg/dl
11. Written consent to participate in the study has been provided by the patient himself/herself.

Exclusion criteria
1. The patient has active multiple cancers (synchronous multiple cancers and metachronous multiple cancers with a disease-free period of ≤5 years.) However, patients with intraepithelial or intramucosal cancer in any organ that is determined to have been cured with localized therapy can be enrolled.
2. In the case of female patients, the patient is possibly pregnant or is breastfeeding.
3. The patient has concurrent interstitial pneumonia, pulmonary fibrosis, or severe emphysema.
4. The patient has concurrent psychiatric disease or psychiatric symptoms determined to make it difficult for the patient to participate in the clinical trial.
5. The patient is receiving continuous systemic steroid therapy (oral or intravenous).
6. The patient has concurrent diabetes mellitus that is being treated with continuous insulin administration or is poorly controlled.
7. The patient has concurrent poorly controlled hypertension.
8. The patient has a history of one or more of the following: serious heart disease, cardiac failure, myocardial infarction within the past 6 months, or angina attacks within the past 6 months.
9. Patients whom the PI or investigators decided as inappropriate for the clinical trial.

ALT, alanine aminotransferase; AST, aspartate aminotransferase; PI, principal investigator.

### Endpoints and sample size calculation

The primary endpoint of COSMOS-CRC-03 will be the 2-year recurrence-free survival (RFS) rate based on Kaplan–Meier curves in patients without detectable ctDNA 28 days post-operatively, which we prospectively defined as the negative predictive value. Based on previous reports,^4^ the 2-year RFS rate with surgery alone for CRC patients with resectable distant metastases is 45%-60%.^3^, ^4^, ^5^ Assuming an expected 2-year RFS value of 70% and a threshold value of 50%, two-sided α of 5%, and power of 80%, the minimum number of ctDNA-negative cases required is 47. Assuming a dropout rate of ∼20%, the target number of ctDNA-negative patients is 60. If the ctDNA-negative rate 28 days post-operatively is assumed to be 40%, the number of patients needed for this trial would be 150.^14^^,^^15^ Furthermore, to account for potential technical errors, we included an additional 10 patients, resulting in a total planned enrollment of 160 patients.

The other primary endpoint is the positive predictive value (PPV) of ctDNA for recurrence during the follow-up period from the completion of curative-intent treatment to 2 years after surgery. Curative-intent treatment is defined as surgery alone for those without ctDNA detected on post-operative day 28 or surgery followed by adjuvant chemotherapy for those with ctDNA detected on post-operative day 28. Assuming that at least 70% of the patients with a positive ctDNA test during the surveillance period will have disease recurrence confirmed by imaging during the observation period,^15^ with a lower threshold of 50%, α of 5% (two-sided), and power of 80%, the required number of patients needed with a positive surveillance test result will be 47. We assume that 60% of the 150 patients who will actually be available for analysis, or 90 patients, will be positive for ctDNA. For patients with a positive ctDNA result after adjuvant chemotherapy (i.e. patients with positive results 28 days after surgery who then received chemotherapy, *n* = 90/160 enrolled), we assume that 50% will have a positive ctDNA result during surveillance (i.e. *n* = 45).^9^ Among the 60 ctDNA-negative patients at 28 days after surgery, we assume that the positive conversion rate within 2 years will be 30% (i.e. *n* = 18). Among them, 50% (i.e. *n* = 9) will test positive after chemotherapy. Therefore, we anticipate a total of 54 patients with a positive ctDNA result during the surveillance period. Considering a dropout rate of 10%, there should be at least 47 relevant patients eligible for PPV analysis.

Secondary endpoints will be as follows: the 2-year RFS rate in patients with ctDNA at 28 days post-operatively; the time from ctDNA detection to recurrence by imaging (lead time for recurrence) during the post-operative surveillance period; the sensitivity of ctDNA for recurrence during the post-operative surveillance period; the percentage of patients positive for ctDNA at each blood collection point; and overall survival (OS). Patients will also undergo ctDNA testing before surgery. However, these results will not be made known to the patient or the attending physician. In the final analysis, a subset analysis will be carried out exclusively on preoperative ctDNA-positive cases. The study is currently ongoing, with planned enrollment through March 2025 and follow-up continuing through March 2027. This study was registered with the Japan Registry of Clinical Trials (jRCT2072220055).

## The aurora trial

### Intervention

This is a randomized controlled trial to evaluate the superiority of mFOLFOXIRI plus bevacizumab (BEV) over mFOLFOX6 therapy in ctDNA-positive patients enrolled in the COSMOS-CRC-03 trial, i.e. patients with detectable ctDNA any time after curative-intent resection of distant CRC metastases (Figure 1). Participating institutions will be expanded to 11 hospitals. Detailed inclusion and exclusion criteria are listed in Table 2. After confirming eligibility, a dynamic allocation using the minimization method will be carried out through the registration allocation system. The stratification factors will be metastatic site (liver only versus other) and primary tumor site location (right versus left). After randomization, the patients will receive either mFOLFOX6 or mFOLFOXIRI + BEV. The schedule of mFOLFOX6 is as follows: 2-h infusion of oxaliplatin 85 mg/m^2^, intravenous leucovorin 200 mg/m^2^ and bolus 400 mg/m^2^ 5-fluorouracil on day 1, and 46-h continuous infusion of 5-fluorouracil 2400 mg/m^2^ every 2 weeks. Up to 12 cycles of this treatment will be administered (24 weeks maximum). The schedule for mFOLFOXIRI + BEV is as follows: 30-90-min infusion of BEV 5 mg/kg, 1.5-h infusion of irinotecan 150 g/m^2^, 2-h infusion of oxaliplatin 85 mg/m^2^, intravenous leucovorin 200 mg/m^2^ on day 1, and 46-h continuous infusion of 5-fluorouracil 2400 mg/m^2^ every 2 weeks. This regimen should be given for up to eight cycles and then followed by up to four additional cycles of 5-fluorouracil plus leucovorin and BEV every 2 weeks (24 weeks maximum).

**Table 2:** AURORA criteria

Inclusion criteria
1. The patient is diagnosed with colorectal cancer based on histological diagnosis of the primary lesion.
2. The patient has participated in the COSMOS-CRC-03 study and has undergone surgical resection of the metastatic lesion.
3. The surgical resection is pathologically diagnosed as R0.
4. ctDNA is positive at 28 days post-operatively or positive during 1-year follow-up period without recurrence during computed tomography/magnetic resonance imaging.
5. Inclusion within 6 weeks after positive ctDNA.
6. Patients will be eligible only if the history of chemotherapy or radiotherapy meets either of the conditions (i) or (ii) below. (i) At the time of enrollment, 28 days have passed since the final infusion of chemotherapy. (ii) Preoperative radiotherapy was administered for the primary or previous metastatic lesion.
7. If metastases to the liver or lungs are present, the patient has no history of cryotherapy or thermocoagulation therapy such as radiofrequency cauterization (including concomitant administration at the time of resection of metastases to the liver).
8. Being aged ≥18 years at the time of obtaining written consent.
9. Eastern Cooperative Oncology Group performance status (PS) of 0 or 1. For patients aged ≥75 years, only a PS 0 is acceptable.
10. The patient has fulfilled all of the following test findings: (The latest test results within the period of 14 days before enrollment should be used. The day 1 week before the enrollment is acceptable.) (i) Neutrophil count ≥1500/mm^3^ (ii) Platelet count ≥10 × 10^4^/mm^3^ (iii) AST ≤100 IU/l (for liver metastases, ≤150 IU/l allowed) (iv) ALT ≤100 IU/l (for liver metastases, ≤150 IU/l allowed) (v) Serum total bilirubin ≤1.5 mg/dl (vi) Cr ≤1.4 mg/dl (vii) Urine protein ≤2+ (viii) Wild-type or single heterozygous (i.e. ∗1/∗6 or ∗1/∗28) UGT 1A1 genotype
11. Written consent to participate in the study has been provided by the patient himself/herself.

Exclusion criteria
1. The patient has active multiple cancers (synchronous multiple cancers and metachronous multiple cancers with a disease-free period of ≤5 years.) However, patients with intraepithelial or intramucosal cancer in any organ that is determined to have been cured with localized therapy can be enrolled.
2. In the case of female patients, the patient is possibly pregnant or is breastfeeding.
3. The patient has concurrent interstitial pneumonia, pulmonary fibrosis, or severe emphysema.
4. The patient has concurrent psychiatric disease or psychiatric symptoms determined to make it difficult for the patient to participate in the clinical trial.
5. The patient is receiving continuous systemic steroid therapy (oral or intravenous).
6. The patient has undergone uncontrolled anticoagulant therapy.
7. The patient with non-healing wound (except for central venous port).
8. Surgery, biopsy with skin incision, and suture procedure for traumatic injury within 28 days before enrollment (except for central venous port).
9. Requiring the continuous treatment of flucytosine, phenytoin, or warfarin potassium.
10. The patient has concurrent diabetes mellitus that is being treated with continuous insulin administration or is poorly controlled.
11. Grade 2 or higher diarrhea or sensory neuropathy.
12. The patient has concurrent poorly controlled hypertension.
13. The patient has a history of one or more of the following: serious heart disease, cardiac failure, myocardial infarction within the past 6 months, or angina attacks within the past 6 months.
14. Patients whom the PI or investigators decided as inappropriate for the clinical trial.

ALT, alanine aminotransferase; AST, aspartate aminotransferase; Cr, creatinine; ctDNA, circulating tumor DNA; PI, principal investigator.

### Endpoints and sample size calculation

The primary endpoint is the proportion of patients without detectable ctDNA at any time point within 12 months after the start of adjuvant chemotherapy. Specifically, this is the proportion of patients who exhibit ctDNA-negative conversion. In addition to ctDNA testing and imaging analysis 28 days after surgery to determine eligibility, patients will have ctDNA testing and imaging at 3, 6, 9, 12 18, and 24 months after surgery. If ctDNA remains positive and the disease recurs, the case will be treated as censored. Once a case converts to negative, it will be considered an event, but follow-up will be continued unless there is a recurrence.

It is expected that 50% of ctDNA-positive patients will become ctDNA negative after post-operative adjuvant chemotherapy including oxaliplatin (unpublished data). Considering that mFOLFOXIRI plus BEV have a 30% higher response rate in first-line therapy than mFOLFOX, we predict a 30% higher ctDNA-negative conversion rate. Based on this prediction, a chi-square test with a one-sided significance level of 5% and a power of 80% yields a target number of 62 patients in both arms. Seventy-four patients (37 per arm) are assigned as the target number, taking into account the number of patients who would be ineligible after enrollment because of the timing of ctDNA results and the start of adjuvant therapy. The secondary endpoints will be RFS, OS, and the time to ctDNA-negative changes. The planned enrollment period for this study is through March 2026 and the follow-up period is through September 2027. This study was registered with the Japan Registry of Clinical Trials (jRCT1071220087).

## Discussion

Perioperative chemotherapy after resection of metastases of CRC is still controversial. The EORTC40983 conducted a randomized controlled study comparing surgical resection alone versus preoperative and post-operative chemotherapy with FOLFOX4 plus surgical resection with curatively resected colorectal liver metastasis (CRLM). A secondary analysis of eligible patients showed a statistically significant difference in the 3-year RFS [29.9% (23.2%-36.9%) versus 39.0% (31.7%-46.3%), respectively, *P* = 0.035], but no significant difference in progression-free survival (PFS) for the primary endpoint of all enrolled patients; moreover, no significant difference was found for OS.^5^ Recently, a randomized phase II/III trial (JCOG0603) of mFOLFOX6 versus surgery alone in patients with CRLM was reported from Japan. The 5-year disease-free survival (DFS) was 38.7% [95% confidence interval (CI) 30.4% to 46.8%] in the surgery-alone group and 49.8% (95% CI 41.0% to 58.0%) in the mFOLFOX6 group, demonstrating a statistically significant improvement. However, the 5-year OS was not statistically improved.^4^

According to these results, the National Comprehensive Cancer Network (NCCN) guidelines suggest chemotherapy during the perioperative period for resection of liver metastases^16^; however, it is not a strong recommendation. Based on these circumstances, it is important to establish evidence of treatment that contributes to improved survival, even though systemic chemotherapy is often used in daily practice after the resection of metastases such as those in the liver and lung. The results of trials completed to date indicate that a uniform approach to chemotherapy for all post-operative patients is unlikely to improve survival, even if drug dosage intensity is increased. In addition to drug dose intensity and duration, we believe that patient stratification is important.

Recently, the relationship between MRD detection using ctDNA and the risk of post-operative recurrence and prognosis in patients with various stages of CRC who have undergone curative surgical resection has been reported. However, no trials have demonstrated the usefulness of ctDNA in this specific setting, namely, CRC with resectable distant metastases. Moreover, no trials have stratified ctDNA-positive patients for more aggressive drug therapy. Our published data show a 50% difference in the 1.5-year DFS between stage IV ctDNA-positive and -negative cases (75.9% and 25.7%, respectively). We therefore believe that stratification by ctDNA is warranted after resection of distant metastases in stage IV disease.^9^ We will investigate the significance of ctDNA stratification in the COSMOS-CRC-03 trial.

Our trial includes a unique randomized phase II trial in which, after resection of ctDNA-positive metastases, patients are considered to have residual tumors and are treated with more aggressive drug therapy. In this AURORA trial, we chose mFOLFOXIRI + BEV as the study arm for adjuvant therapy. BEV has not been shown to be effective for adjuvant therapy in stage II-III patients. Patients who have undergone resection of ctDNA-positive metastases frequently experience relapse and may already have micrometastases that are not detectable by imaging studies. Therefore, the high response rate of this treatment provides a rationale for its use as the study arm. In addition, one endpoint of the study was the ctDNA-negative conversion rate. Because all patients included in the trial will be ctDNA positive, the ctDNA-negative conversion rate may provide a means to quickly obtain results. It would serve as a useful advanced endpoint in this study. However, it is still unknown if the negative conversion rate of ctDNA can be used as an endpoint. Thus, we must ascertain whether negative conversion correlates with PFS or OS.

These attempts will help stratify the treatment of patients who undergo resection of distant metastases and will further contribute significantly to improving the prognosis of stage IV CRC.

## Ethics approval and consent to participate

The COSMOS-CRC-03 and AURORA trials are conducted in accordance with the Declaration of Helsinki, the Japanese Ethical Guidelines for Medical and Health Research Involving Human Subjects, and the Clinical Trial Acts in Japan. Each trial was approved by the Kyushu University Hospital Certified Review Board. Written informed consent was obtained from all patients before their enrollment.
